# Supplementary figures and images for: Ecotin protects Salmonella Typhimurium against the microbicidal activity of host proteases
Source: PLoS Pathog. 2025 Mar 28;21(3):e1013013. doi: 10.1371/journal.ppat.1013013 (PMC11977995; doi:10.1371/journal.ppat.1013013)

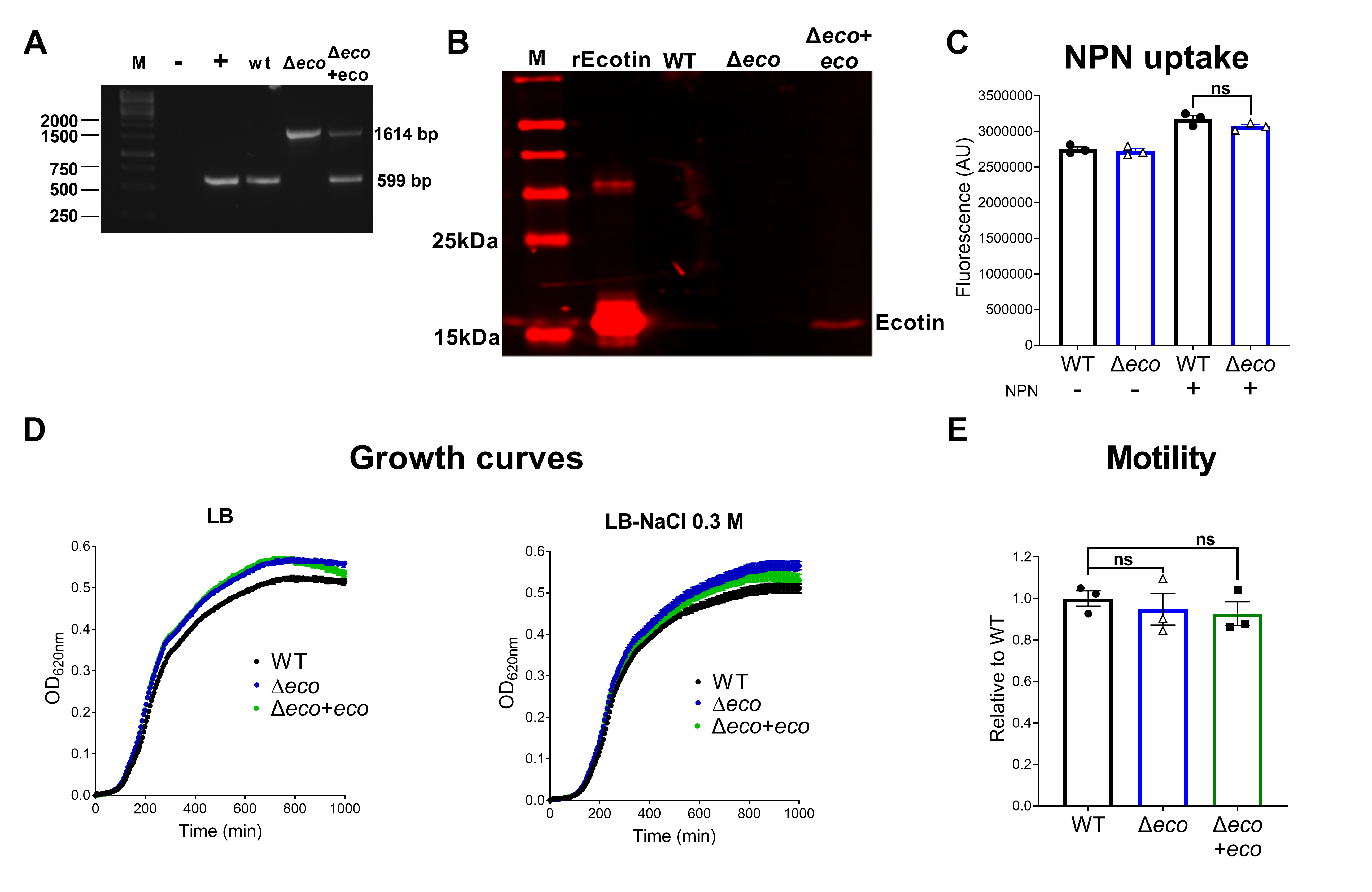

Supplement: S1 Fig — (A) PCR made with the specific primers flanking the ecotin (EcotinFP and EcotinRV) gene showing a band of 599 bp where ecotin is present and a band of 1614 bp where the kanamycin resistance cassette is present. The positive control (+) corresponds to STm genomic DNA as template. For the WT, ∆eco and ∆eco+eco strains, one fresh colony was used for colony-PCR. (B) Western blot of bacterial lysates for each strain stained with mouse anti-Ecotin primary antibody and secondary rabbit anti-mouse. Recombinant Ecotin was added as a positive control. (C) Study of influence of ecotin on the membrane permeability of STm using N-phenyl-1-naphthylamine (NPN) uptake assay. Fluorescence was plotted after 10min incubation. Bars represent the mean±SEM. Dots represent independent experiments. Student’s t-test. nsp>0.05. (D) Analysis of influence of ecotin in growth curves. Bacteria from overnight cultures were diluted in LB or LB-NaCl 0.3M and seeded in 96 wells plate to measure OD620nm using a microplate reader at 37°C and agitation. Representative of three experiments. (E) Swimming assays performed to assess possible contribution of ecotin to motility. A 2µL spot of overnight cultures was seeded in the middle of a LB-Agar 0.3% plate. Then the plates were incubated at 30°C for 24h. The area was normalized by the WT strain. Dots represents independent experiments. Bars represent the mean±SEM. One-way ANOVA with Bonferroni post-test. nsp>0.05. (TIF) [file ppat.1013013.s002.tif]

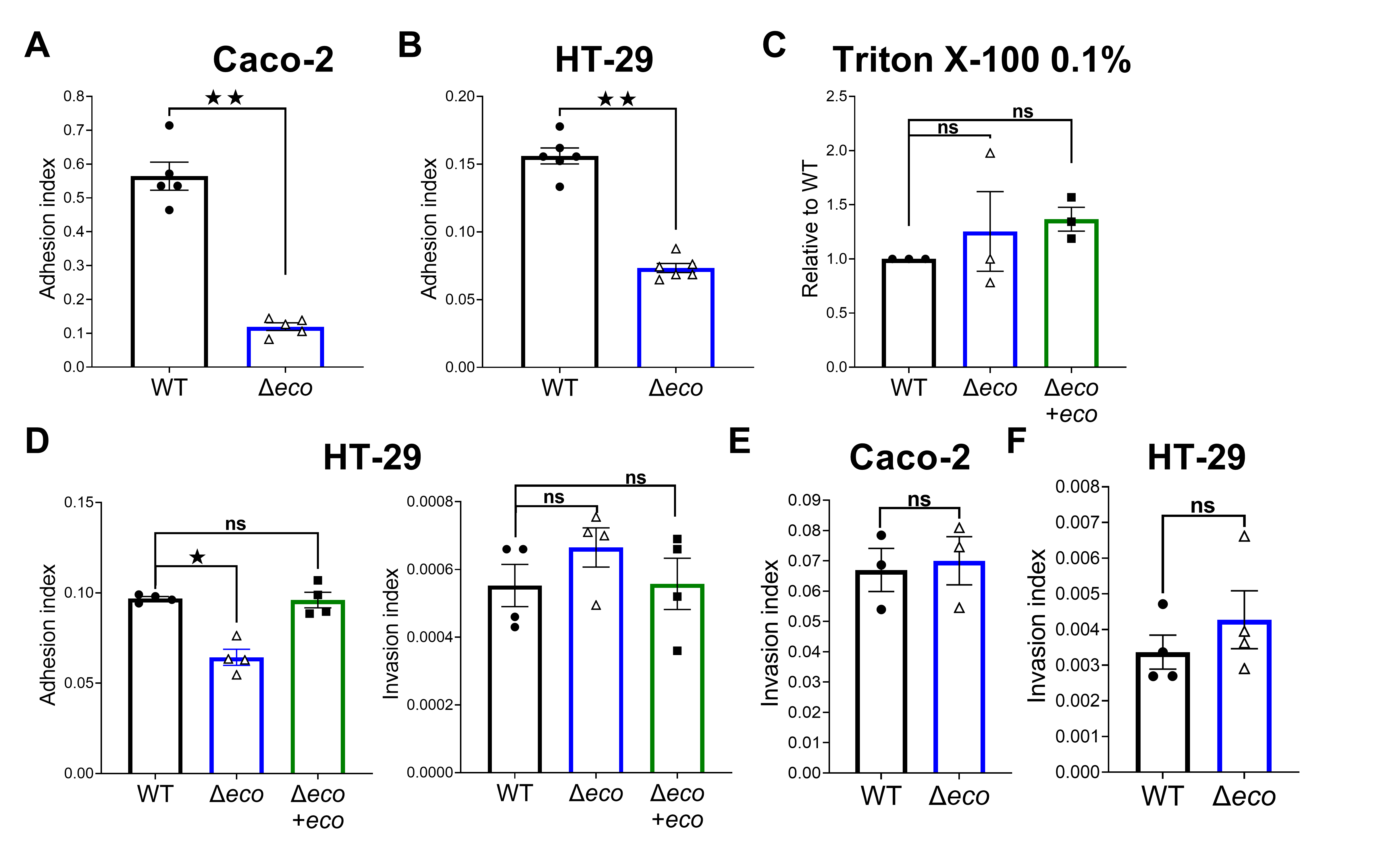

Supplement: S2 Fig — (A-B) Adhesion assays in Caco-2 and HT-29 monolayers with centrifugation of bacteria and monolayers for 5min at 400xg before the 30min incubation. Bars represent the mean±SEM. Dots are technical replicates. Data from one representative experiment of at least two independent experiments. Student’s t-test. **p<0.01. (C) 106 CFU of the WT, ∆eco or ∆eco+eco strains were incubated with Triton X-100 0.1% to study if they were susceptible to its action. After 15min at room temperature, serial dilutions and plating were performed to assess bacterial counts and then they were normalized by the WT strain. Bars represent the mean±SEM. Dots represent independent experiments. Kruskal-Wallis test. nsp>0.05. (D) Adhesion and invasion assay in HT-29 monolayers. To measure invasion, the monolayers were incubated 30 min with the indicated strains and then further incubated with gentamicin 100 µg/mL for 60 min before performing bacterial counts. The invasion index was calculated as the bacteria recovered after gentamicin normalized to the bacteria in the inoculum. Bars represent the mean±SEM. Dots are technical replicates. Data from one experiment. Kruskal-Wallis test. nsp>0.05, *p<0.05. (E-F) Invasion assay in Caco-2 and HT-29. Similar to (D) but using 90 min incubation instead of 30 min before adding the gentamicin to kill extracellular bacteria. The invasion index was calculated as the bacteria recovered after gentamicin normalized to the bacteria in the inoculum. Bars represent the mean±SEM. Dots are technical replicates. Data from one representative experiment of two independent experiments. Student’s t-test. nsp>0.05, *p<0.05. (TIF) [file ppat.1013013.s003.tif]

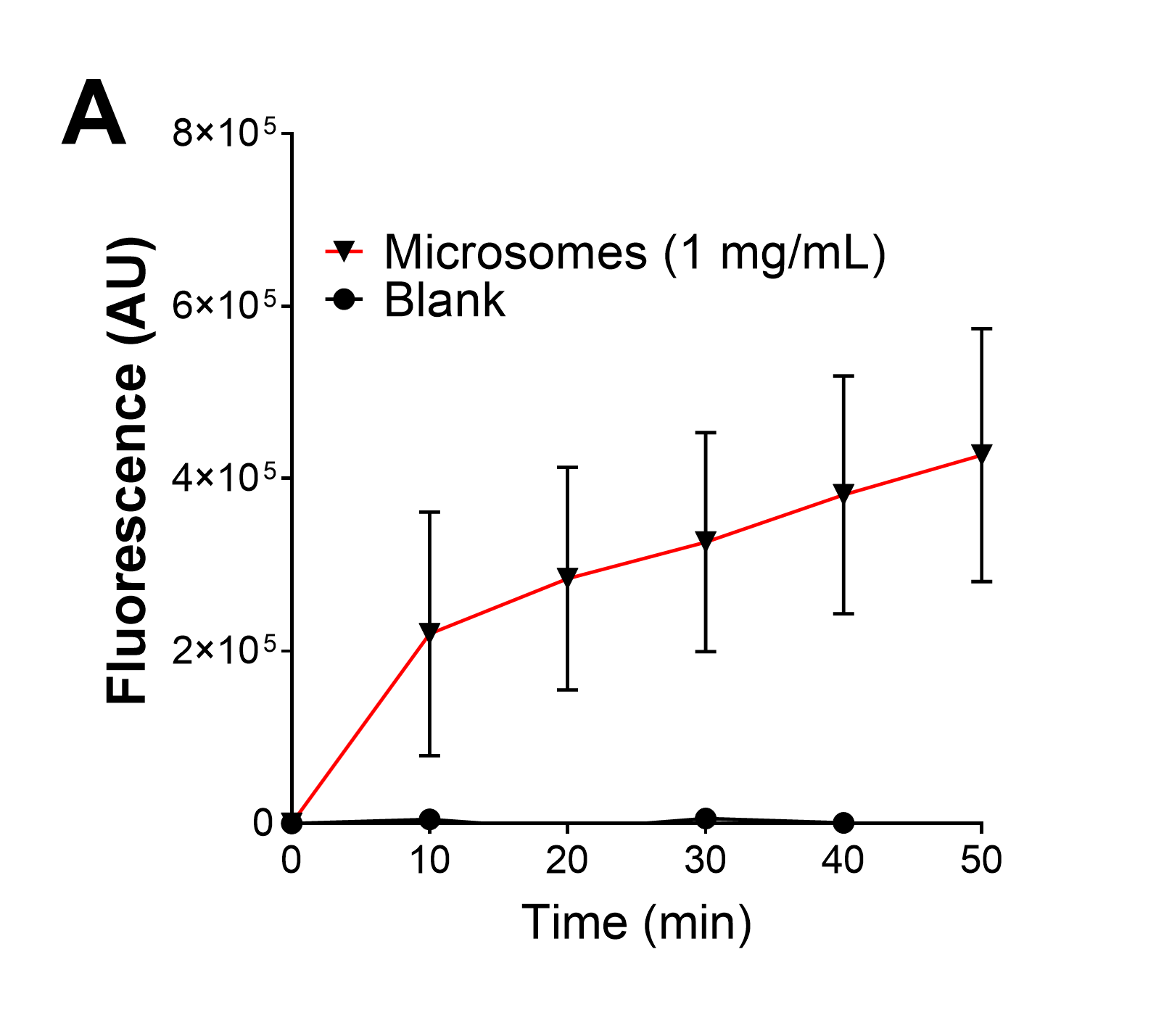

Supplement: S3 Fig — (A) Microsomes at a concentration of 1 mg/mL were co-incubated with casein-BODIPY and fluorescence was measured over the time. Dots are mean±SEM. Data from one representative experiment of at least two independent experiments. (TIF) [file ppat.1013013.s004.tif]

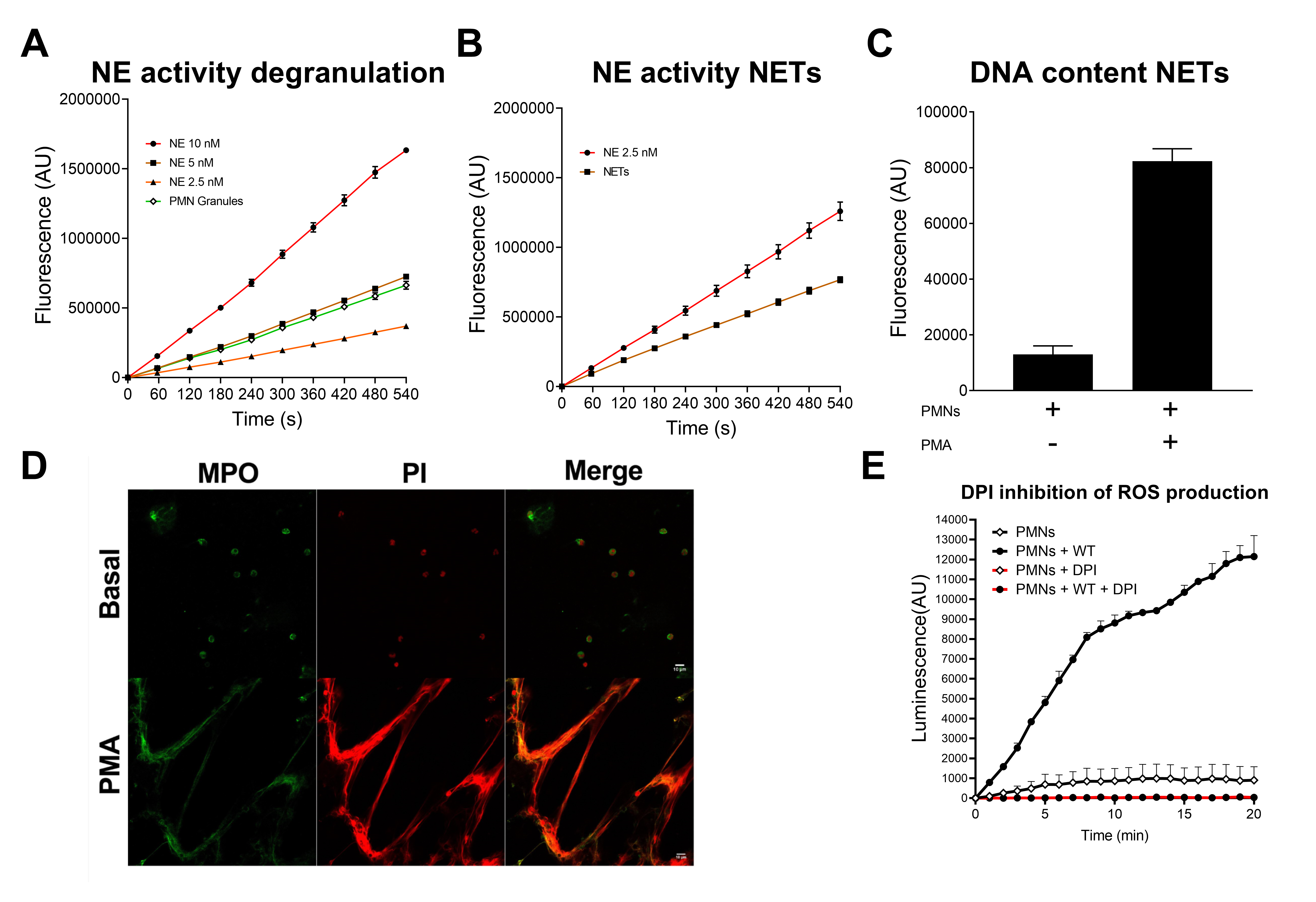

Supplement: S4 Fig — (A) Degranulated supernatants were co-incubated with neutrophil elastase specific fluorescent substrate and fluorescence was measured over the time. Purified neutrophil elastase was used as control in the indicated concentrations. Dots are mean±SEM. Data from one blood donor. (B) NETs were co-incubated with neutrophil elastase specific fluorescent substrate and fluorescence was measure over the time. Purified neutrophil elastase was used as control in the indicated concentration. Dots are mean±SEM. Data from one blood donor. (C) DNA determination by Sytox Green addition, the more DNA the more fluorescence obtained. The fluorescence was measured in a microplate reader. Bars are mean±SEM. Data from one blood donor. (D) Purified human PMNs were seeded in glass coverslips pre-treated with poly-L-lysine. Then, PMNs were left untreated (Basal) or treated with PMA 100 nm for 4 h (PMA), neutrophil myeloperoxidase (MPO) was stained with a green fluorescent primary antibody and DNA was stained with propidium iodide (IP). Confocal microscopy images from a representative field are shown showing NETs formation after PMA treatment. (E) To assess the ability of DPI to inhibit ROS production a luminol assay was used. Luminol emits luminescence in the presence of ROS, thus measuring the luminescence intensity in a microplate reader serves as an indicator of ROS formation. Neutrophils were pre-treated or not with DPI 10 µM for 20 min before co-incubating with a MOI 1:1 of STm WT strain. Each point represents a time point. The mean data of 2 independent donors is shown. (TIF) [file ppat.1013013.s005.tif]

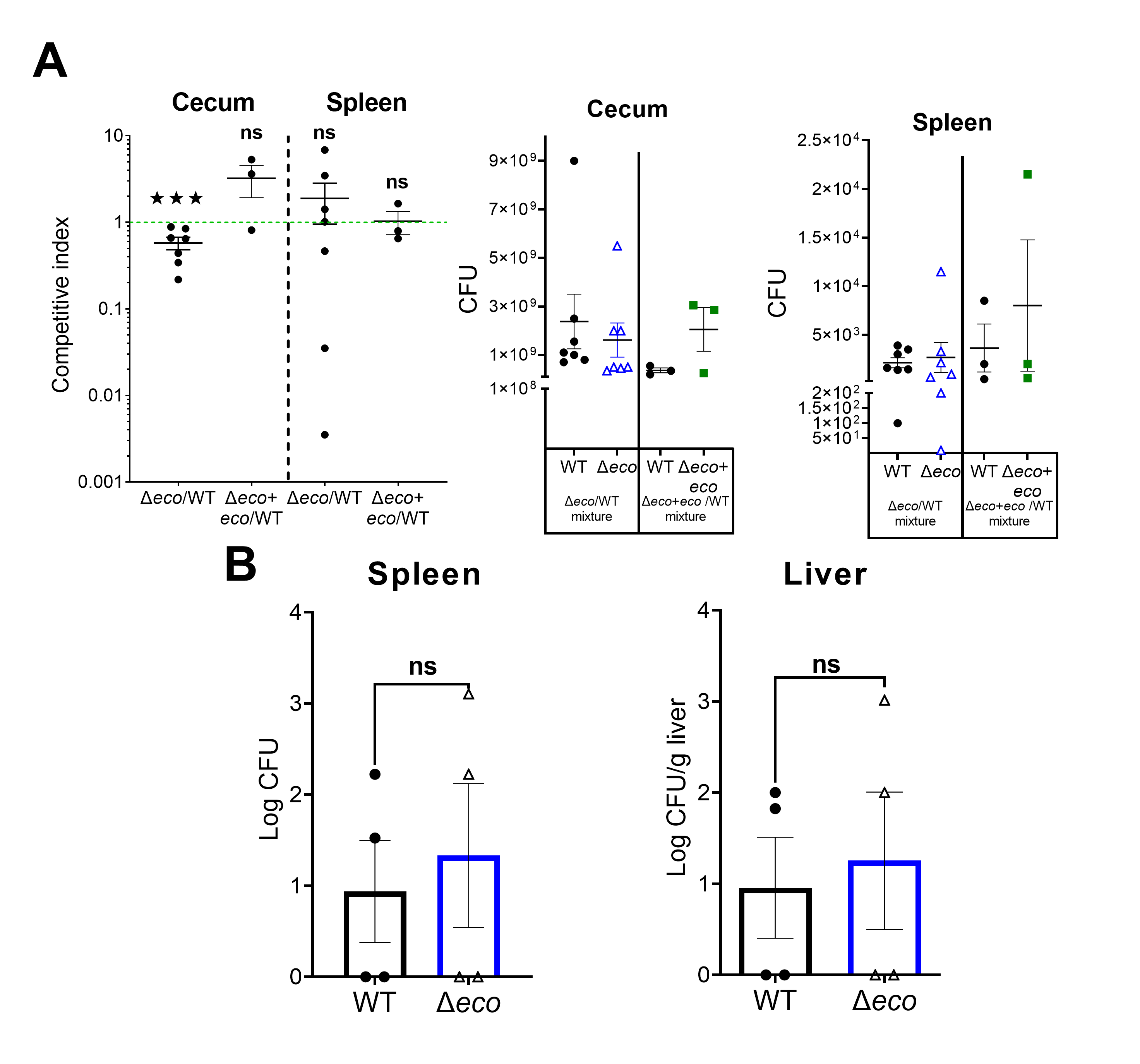

Supplement: S6 Fig — (A) Streptomycin pre-treated mice were given a 1:1 mixture of WT:Δeco (n=6) or WT:Δeco+eco (n=3) by oral gavage containing 107 CFU (n=6) or WT and Δeco+eco (n=3). At 72 h p.i. the animals were sacrificed and the cecum and spleen were harvested. The organs were processed to determine the bacterial load using agar-SS plates with or without antibiotics to determine the ratio Δeco/WT or Δeco+eco/WT. The ratios were normalized to the ratio in the inoculum to obtain the competitive index (left panel). The actual CFU recovered from cecum and spleen are shown in the middle and right panel respectively. The mean±SEM is presented. Each point represents an individualmouse. One sample T-test vs 1. nsp>0.05, ***p< 0.001 (B) Mice were given 105 CFU of WT (n=4) or Δeco (n=4) strains and 120 h later they were sacrificed to assess dissemination. The spleen and a portion of the liver were harvested and processed to determine the bacterial load. Bacterial load was normalized per spleen and per gram of liver respectively. The bars represent mean±SEM. Each point represents an individual mouse. Mann-Whitney test. nsp>0.05. (TIF) [file ppat.1013013.s007.tif]

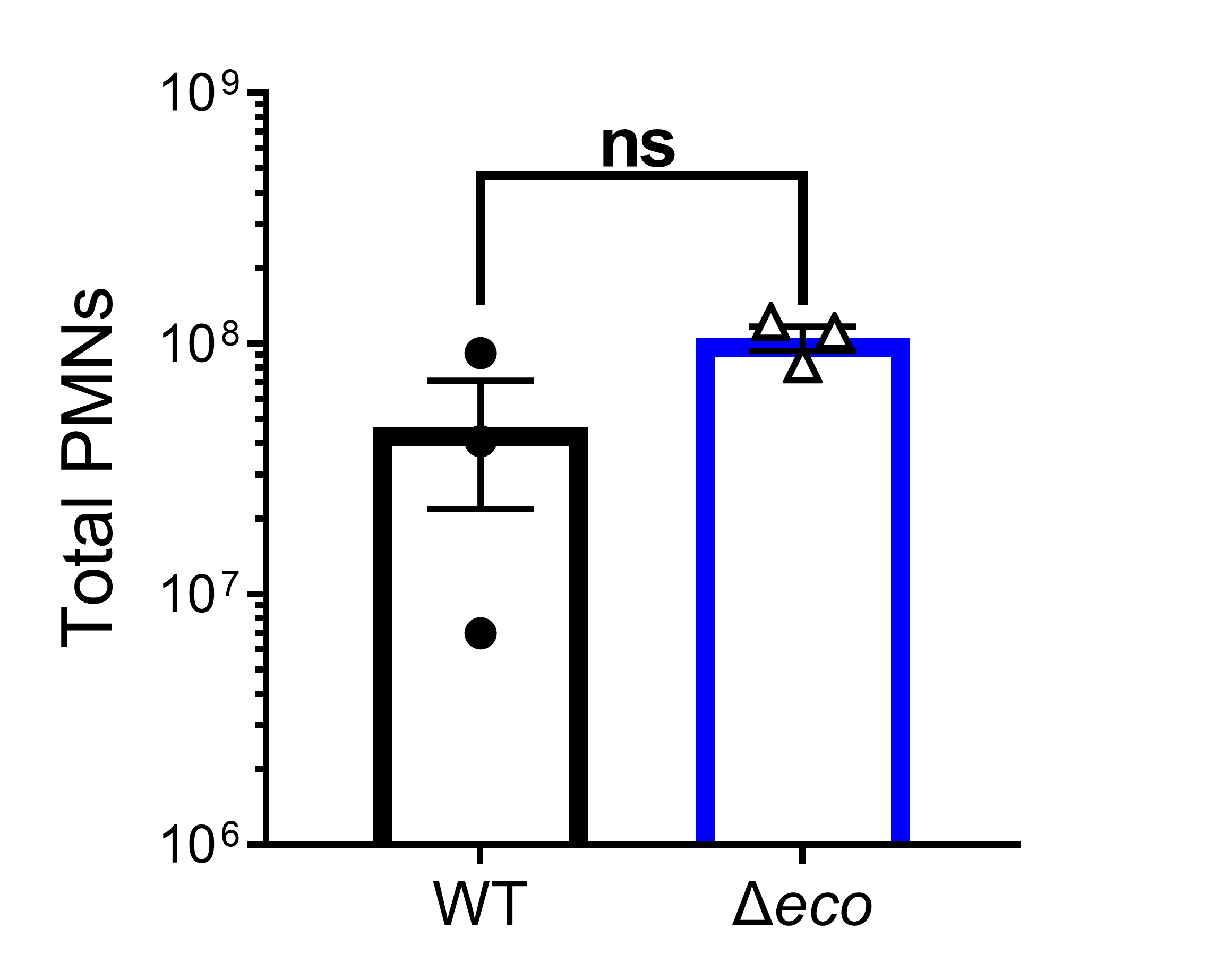

Supplement: S7 Fig — (A) BALB/c mice were given by intraperitoneal injection 104 CFU of WT (n=3) or Δeco (n=3) strains. Then, 4 h after infection, mice were sacrificed and suspended cells obtained by peritoneal lavage with RPMI. The cells were labeled with anti-Ly6G, anti-CD11b and anti-Ly6C, the triple positive cells were considered PMNs. The bars represent mean±SEM. Each point represents an individual mouse. Mann-Whitney test. nsp>0.05. (TIF) [file ppat.1013013.s008.tif]
