# Supplementary material for: Ecotin protects Salmonella Typhimurium against the microbicidal activity of host proteases
Source: PLoS Pathog. 2025 Mar 28;21(3):e1013013. doi: 10.1371/journal.ppat.1013013 (PMC11977995; doi:10.1371/journal.ppat.1013013)
Supplement: S1 Table — (PDF) [file ppat.1013013.s009.pdf]

Supplementary Table 1. Strains, Plasmids and primers

Strains	Description	Source
14028s	*Salmonella* Typhimurium wild type	ATCC
Δ*ecotin*	*Salmonella* Typhimurium with the *ecotin* gene replaced by a kanamycin cassette	[1]
Complemented strain (Δ*eco*+*eco*)	Δ*ecotin* mutant harboring the pWSK29 plasmid which expresses the *ecotin* gene	This study
**Plasmids**	**Description**	**Source**
pWSK29	Plasmid used for cloning *ecotin* gene	[2]
pBR322-Timer	Plasmid used as a bacterial growth reporter	[3]
pNCS-mClover3	GFP expressing plasmid	[4]
**Primers**	**Sequence**	**Source**
EcotinFPcomple	AACAAGCTTCAGTTGCCCGCTTTCAACAT	This study
EcotinRVcomple	AACTCTAGATCACTGACGTTGCTGTTCCT	This study
EcotinFP	TCTGTTGTCAGTGAATACCGAAA	This study
EcotinRV	GTGAGTCGGCGTGTTCAGA	This study

REFERENCES

1. Porwollik, S., et al., *Defined single-gene and multi-gene deletion mutant collections in Salmonella enterica sv Typhimurium.* PLoS One, 2014. **9**(7): p. e99820.
2. Wang, R.F. and S.R. Kushner, *Construction of versatile low-copy-number vectors for cloning, sequencing and gene expression in Escherichia coli.* Gene, 1991. **100**: p. 195-9.
3. Claudi, B., et al., *Phenotypic variation of Salmonella in host tissues delays eradication by antimicrobial chemotherapy.* Cell, 2014. **158**(4): p. 722-733.
4. Bajar, B.T., et al., *Improving brightness and photostability of green and red fluorescent proteins for live cell imaging and FRET reporting.* Sci Rep, 2016. **6**: p. 20889.
